# The Role of COVID-19 Pandemic Anxiety and Perceptions in COVID-19 Vaccination

**DOI:** 10.1192/j.eurpsy.2023.1667

**Published:** 2023-07-19

**Authors:** E. R. Semenova, J. Konyukhovskaya, S. Sviridova, E. Pervichko, O. Stepanova, O. Mitina, I. Shishkova

**Affiliations:** 1 Moscow State University named after ‘M. V. Lomonosov’; 2 Pirogov Russian National Research Medical University, Moscow; 3 Ryazan State Medical University, Ryazan, Russian Federation

## Abstract

**Introduction:**

Since stress and anxiety are significant manifestations of psychological distress during the COVID-19 pandemic, we studied their role in making a decision about vaccination.

**Objectives:**

To study the relationship between the intention to be vaccinated against COVID-19 with health anxiety and stress levels.

**Methods:**

The methodological complex includes the author’s socio-demographic questionnaire (Pervichko, 2020, 2021, 2022); the questionnaire “Scale of perceived stress-10” (Ababkov, 2016); the questionnaire “Perceptions of the COVID-19 pandemic” (Pervichko et al., 2020), developed on the basis of the Russian-language version of the E. Broadbent’s short questionnaire about the perception of disease (Broadbent, 2006); the State-Trait Anxiety Inventory (STAI) (Spielberger, 2002) and the “Short Health Anxiety Inventory” (Pervichko et al., 2020).

The study involved 232 respondents who did not have COVID-19 (average age – 29.1 ± 13.7 years). Among the respondents, 68.5% have already been vaccinated, 23.3% do not plan to be vaccinated and 8.2% plan to perform the procedure.

**Results:**

The methodological complex includes the author’s socio-demographic questionnaire (Pervichko, 2020, 2021, 2022); the questionnaire “Scale of perceived stress-10” (Ababkov, 2016); the questionnaire “Perceptions of the COVID-19 pandemic” (Pervichko et al., 2020), developed on the basis of the Russian-language version of the E. Broadbent’s short questionnaire about the perception of disease (Broadbent, 2006); the State-Trait Anxiety Inventory (STAI) (Spielberger, 2002) and the “Short Health Anxiety Inventory” (Pervichko et al., 2020).

The study involved 232 respondents who did not have COVID-19 (average age – 29.1 ± 13.7 years). Among the respondents, 68.5% have already been vaccinated, 23.3% do not plan to be vaccinated and 8.2% plan to perform the procedure.

**Conclusions:**

Higher health anxiety, situational anxiety, perceived stress, and greater perceived life threat due to coronavirus contribute to COVID-19 immunization procedures, which is accompanied by perceptions of greater control of the pandemic.

**Disclosure of Interest:**

None Declared

